# FACTOR STRUCTURE OF THE BRIEF PAIN INVENTORY–SHORT FORM IN AFRICAN AMERICAN OLDER ADULTS WITH OSTEOARTHRITIS

**DOI:** 10.1093/geroni/igad104.2802

**Published:** 2023-12-21

**Authors:** Jianli Wu, Ann Horgas, Staja Booker

**Affiliations:** University of Florida, Gainesville, Florida, United States; University of Florida, Gainesville, Florida, United States; University of Florida, Gainesville, Florida, United States

## Abstract

The Brief Pain Inventory-Short Form (BPI-SF) is a widely used questionnaire designed to assess aspects of cancer pain, such as pain intensity and pain interference. Psychometric properties have rarely been evaluated in other pain conditions such as osteoarthritis. Osteoarthritis is a major contributor to pain and physical disability in African Americans (AA) older adults. Thus, it is important to evaluate the psychometric properties of BPI-SF in this health disparity population. Participants were AA aged 50-94 years with self-reported osteoarthritis. The final convenience sample (N=110) completed surveys including a demographics questionnaire and the validated BPI-SF. Confirmatory Factor Analysis (CFA) was used to evaluate the factor structure. Measurement invariance across two age groups [i.e., young-old (50-69); old-old (70-94)] was examined. CFA revealed that a three-factor model (i.e., pain intensity, activity interference, and affective interference) demonstrated the best fit (χ2/df=1.595, CFI=0.949, RMSEA=0.074) to the data. AAs reported pain intensity (M=4.58, SD=2.01), affective interference (M=3.03, SD=2.54), and activity interference (M=4.19, SD=3.25). The change of CFI between configural and metric invariance is below the cutoff point of .01, supporting the full metric (i.e., factor loadings) invariance across two age groups. However, the full scalar (i.e., items intercepts) invariance was not established. The results support a three-factor structure of BPI-SF that is invariance across two age groups at the full metric level. This suggests that the BPI-SF is an appropriate measure to characterize chronic pain intensity, affective interference, and activity interference for older AAs with osteoarthritis.

